# Health disparities in aging: Improving dementia care for Black women

**DOI:** 10.3389/fnagi.2023.1107372

**Published:** 2023-02-09

**Authors:** Caleigh A. Findley, MaKayla F. Cox, Adam B. Lipson, RaTasha Bradley, Kevin N. Hascup, Carla Yuede, Erin R. Hascup

**Affiliations:** ^1^Department of Neurology, Dale and Deborah Smith Center for Alzheimer’s Research and Treatment, Neuroscience Institute, Southern Illinois University School of Medicine, Springfield, IL, United States; ^2^Department of Pharmacology, Southern Illinois University School of Medicine, Springfield, IL, United States; ^3^Department of Neurology, Washington University, St. Louis, MO, United States; ^4^Division of Neurosurgery, Department of Surgery, Southern Illinois University School of Medicine, Springfield, IL, United States; ^5^Medical Microbiology, Immunology and Cell Biology, Southern Illinois University School of Medicine, Springfield, IL, United States; ^6^Department of Psychiatry, Washington University, St. Louis, MO, United States

**Keywords:** Alzheimer’s disease, Alzheimer’s disease and related dementias, discrimination, outreach, healthcare equity

## Abstract

In the United States, 80% of surveyed Black patients report experiencing barriers to healthcare for Alzheimer’s disease and related dementias (ADRD), delaying the time-sensitive treatment of a progressive neurodegenerative disease. According to the National Institute on Aging, Black study participants are 35% less likely to be given a diagnosis of ADRD than white participants, despite being twice as likely to suffer from ADRD than their white counterparts. Prior analysis of prevalence for sex, race, and ethnicity by the Centers for Disease Control indicated the highest incidence of ADRD in Black women. Older (≥65 years) Black women are at a disproportionately high risk for ADRD and yet these patients experience distinct inequities in obtaining clinical diagnosis and treatment for their condition. To that end, this perspective article will review a current understanding of biological and epidemiological factors that underlie the increased risk for ADRD in Black women. We will discuss the specific barriers Black women face in obtaining access to ADRD care, including healthcare prejudice, socioeconomic status, and other societal factors. This perspective also aims to evaluate the performance of intervention programs targeted toward this patient population and offer possible solutions to promote health equity.

## 1. Introduction

**Figure 1:**
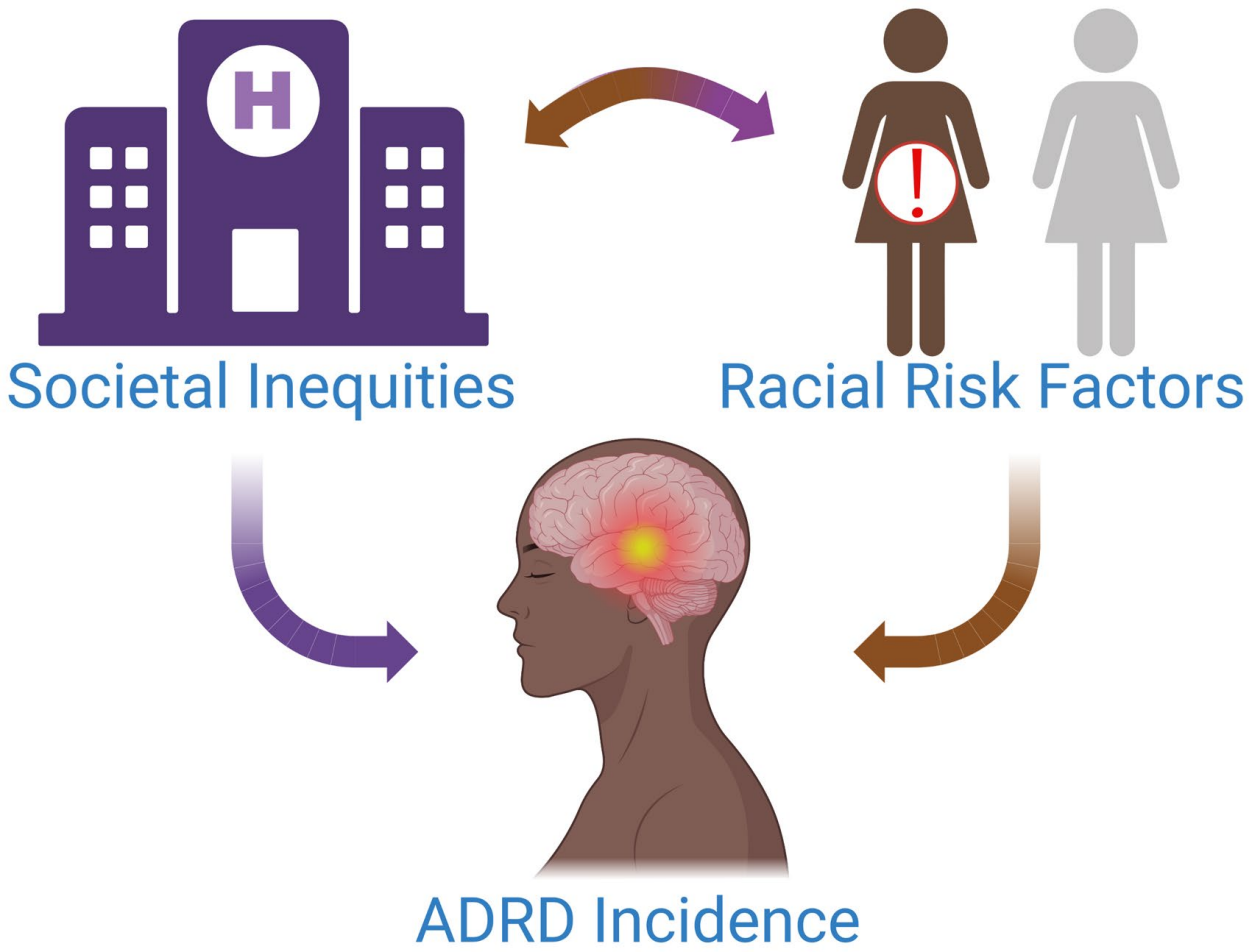
Multiple factors contribute to the increased incidence of ADRD in Black women. Societal inequities and discrimination drive chronic stress and inhibit access to high-quality healthcare for this patient population. Over time, this can have serious biological and health implications for patients, placing them at an elevated risk for modifiable diseases associated with ADRD and potentiating their risk for ADRD.

“It’s a social justice and bioethics issue,” Carl V. Hill, Ph.D., MPH, Chief Diversity, Equity, and Inclusion Officer for the Alzheimer’s Association, remarks while reflecting on the current state of dementia care for Black women. Having established the framework for health disparities at the National Institute on Aging (NIA) before joining the Alzheimer’s Association, Hill is no stranger to tackling some of the toughest problems in health equity (Hill et al., 2015). The current state of affairs in Alzheimer’s disease and related dementias (ADRD) care for Black patients, particularly Black women, resides toward the top of such a list. As the fifth leading cause of death in older Black Americans, ADRD poses a great risk to patient health in this demographic (Us Against Alzheimer’s, 2021). Current estimates indicate that Black individuals are at 2–3 times greater risk of developing AD than their White counterparts with the highest risk for those with familial history of AD (Green et al., 2002; Younan et al., 2022). Black Americans could face up to four times the amount of ADRD cases over present-day estimates by 2060, as the aging population balloons to more than double by 2030 (Matthews et al., 2019; Alzheimer’s Association, 2022). As such, the elevated risk of ADRD for Black individuals will only continue to worsen in the coming years (Figure 1).

Women are approximately twice as likely to develop ADRD than men across all races and ethnicities (Lim et al., 2022). The CDC found the highest prevalence in women and Black individuals when examining ~3.2 million Medicare Fee-For-Service beneficiaries diagnosed with ADRD in 2014 (Matthews et al., 2019). Research examining age-standardized diagnostic incidence rate and relative risk of late-onset ADRD also found the highest risk for Black women (Lim et al., 2022). Black women live at the crossroads of the most vulnerable populations for ADRD. Yet, research efforts tend to focus more broadly on either race or sex, giving little credence to the compounding effect that has led Black women to have the highest amount of ADRD cases. To that end, this perspective article will review the current understanding of contributing factors for increased ADRD risk in Black women, including ADRD healthcare access barriers, and evaluate current interventional programs and strategies to address this issue.

## 2. Chronic discrimination as a neurobiological insult in black patients

The biological and environmental pathways that lead to poorer health outcomes in Black patients are complex and intertwined. Researchers have long established that extended periods of stress can wreak havoc on patient’s physical and mental health and brain function (Kloet et al., 2005; Blair and Cybele Raver, 2016). A lifetime of exposure to systemic racism and discrimination for Black patients places them in a vulnerable biological standing due to chronic psychological stress and impacts to mental health (Geronimus, 1992; Geronimus et al., 2010; Blair and Cybele Raver, 2016). Anxiety, depression, and other psychiatric conditions can increase risk for ADRD and negatively impact patient health outcomes (Richmond-Rakerd et al., 2022). Prior studies show that Black Americans have a higher allostatic load, referring to the cumulative damage done to the body under chronic stress. Black women had the highest allostatic load scores compared to Black men and White persons, regardless of income level (Geronimus et al., 2006). Evidence may also support that Black women between 49 and 55 years of age are roughly 7.5 years older biologically than their White counterparts, as assessed by telomere length – a repetitive sequence of DNA found at the end of chromosomes – from peripheral blood mononuclear cells (Geronimus et al., 2010). These sequences are used to protect chromosomes during cell division and replication. Telomere length is a well-established biomarker with an inverse relationship to age, as telomeres shorten throughout the lifespan (Houben et al., 2008). Stress pathways chronically activated by psychosocial stressors can increase the rate of telomere shortening and, in turn, indicate biological aging (Epel et al., 2004; Cherkas et al., 2006; Rewak et al., 2014). Data support longer telomere length at birth in Black patients than White patients, with accelerated shortening observed most specifically in Black women (Roux et al., 2009; Rewak et al., 2014; Brown et al., 2016).

The biological consequences of discrimination for Black patients are also observed in ADRD. The onset of ADRD symptoms typically occurs at an earlier age and with greater severity for Black Americans compared to their White counterparts (Barnes and Bennett, 2017). Black Americans with high brain amyloid deposition showed a more severe cognitive decline over a 20-year follow-up than White patients with similar amyloid load (Gu et al., 2015; Barnes and Bennett, 2017; McDonough, 2017). Similar studies demonstrate that Black patients with high brain amyloid exhibit smaller hippocampal volumes and decreased cortical thickness compared to matched White patients (McDonough, 2017). As participants were matched on several parameters, these findings may indicate a faster neurodegenerative rate in Black patients. Recent studies have focused on biological mechanisms involved in both stress and ADRD, such as inflammation and oxidative damage pathways. One pilot study demonstrated an association between high interleukin-10 levels and impaired executive function but yielded mixed results on cognitive measures (Patel et al., 2020). Reduced AD risk in Black women that utilize statins has also been observed, possibly supporting that metabolic dysfunction and inflammation act as primary biological drivers for increased ADRD incidence in Black women (Zissimopoulos et al., 2017). The elevation of these driving factors is in line with previously reported chronic stress and cardiovascular disease in this patient population (Stampfer, 2006; Geronimus et al., 2010; Centers for Disease Control and Prevention, 2018). However, pilot studies such as these are limited by their sample sizes and possible sampling bias due to the lack of recruitment of Black patients in ADRD research (Barnes and Bennett, 2017). Current knowledge on the driving force of neurological vulnerability and possible accelerated biological aging in Black women remains limited and warrants further study.

## 3. Institutional discrimination drives ADRD health disparity in black women

Joyce Balls-Berry, Ph.D., Associate Professor at Washington University in St. Louis and the Health Disparities and Equity Core at the Knight Alzheimer’s Disease Research Center (ADRC), emphasizes that “one of the big problems that we have, especially for women of color and even just communities of color regardless of self-identity on race or ethnicity, is a delay in diagnosis.” Several barriers contribute to this delay and prevent Black patients from accessing the highest quality care, including healthcare prejudice and disparities, lack of culturally competent care, and systemic racism.

Only 20% of surveyed Black Americans feel they have no barriers to quality healthcare for ADRD (Alzheimer’s Association, 2021). The highest levels of discrimination in dementia health care are experienced by Black Americans, according to two national surveys from the Alzheimer’s Association (Alzheimer’s Association, 2021). Healthcare prejudice impacts all facets of care and can have serious implications for the health of Black patients with ADRD. A recent study from the NIA found that Black participants were 35% less likely to receive a diagnosis than White participants, despite having a well-established higher incidence of ADRD (Lennon et al., 2022). Black patients with an ADRD diagnosis are also less likely to have been told by a doctor that they have a “memory-related disease” (Alzheimer’s Association, 2021). These findings support possible underdiagnosis of ADRD in Black patients and may have larger implications for the current understanding of ADRD incidence in this patient population.

Disparities in patient treatment for modifiable risk factors of ADRD may contribute to their increased incidence for Black women. Obesity, diabetes, and hypertension are all modifiable risk factors for ADRD (Omura et al., 2022). Black women experience a greater incidence of all three conditions than Black men and White individuals (Agyemang and Powell-Wiley, 2013). In the United States, Black women are 70% more likely to experience obesity in their lifetime than White women (Agyemang and Powell-Wiley, 2013; Williams et al., 2015). The increased incidence of obesity in Black women likely also contributes to their disproportionate rate of diabetes. An estimated 1 in 4 Black women (≥ 55 years old) has diabetes, occurring at roughly twice the rate of White women (Beckles and Thompson-Reid, 2001; Agency for Healthcare Research and Quality, 2016; Centers for Disease Control and Prevention, 2022). Diabetes and obesity tend to co-occur with hypertension, which affects 58% of Black women in the U.S. compared to only 41% of White and Hispanic women (Virani et al., 2021). Almost half (46%) of Black women suffer from the highest level of hypertension (Stage 2), comprising a greater portion of the population than Black men (42%) (Abel et al., 2021). Hypertension is also a major risk factor for cardiovascular disease, the leading cause of death in Black women, and a significant risk factor for ADRD (Stampfer, 2006; Centers for Disease Control and Prevention, 2018). The higher prevalence of these conditions and discrimination in health care, especially for Black female patients, likely contribute to their unequal burden of ADRD prevalence.

Healthcare discrimination against Black patients extends beyond the scope of ADRD and has implications for patient health outcomes. Patients of racial and ethnic minorities in the U.S. are less likely to receive preventative healthcare and are more likely to experience a lower quality of care (Smedley et al., 2003; Institute of Medicine, 2012; Agency for Healthcare Research and Quality, 2021). The same report showed that Black patients’ health outcomes remain worse than for White patients even when accounting for income, neighborhood status, comorbid illnesses, and health insurance type. One study also demonstrated a strong link between healthcare discrimination based on race, sex, age, education, or income level and the health outcomes of diabetic patients (Piette et al., 2006). Black patients are less likely to receive recommended services for diabetes and less likely to have their hemoglobin A1c and blood pressure under control (Millett et al., 2008; Satcher, 2008; Agency for Healthcare Research and Quality, 2016). The unequal treatment of Black patients could have implications for health outcomes long before their ADRD diagnosis.

Tantamount to discrimination is a lack of access to culturally competent care for Black ADRD patients. Only 48% of surveyed Black persons report feeling confident they can access culturally competent care (Alzheimer’s Association, 2021). Their fears are not unfounded, as a growing amount of evidence supports that common diagnostic tools are less accurate for non-White patients. Many cognitive tests commonly utilized in ADRD clinical evaluation, like the Stroop Color and Word Test or the Mattis Dementia Rating Scale, include race-norming adjustments that assume lower performance scores for Black patients and thus complicate the interpretation of low performance scores that may result from cognitive impairment (Gasquoine, 2009; Vyas et al., 2020; Barnes, 2022). Race-norming in cognitive tests may prevent Black patients experiencing dementia symptoms from getting the proper diagnosis. Other cognitive tests included in the ADRD battery, such as the Mini-Mental State Exam, are known to suffer from racial bias that results in the misdiagnosis of Black patients. These neuropsychological tests can yield staggering false-positive results between races and ethnicities, up to 42% in Black patients compared to 6% in their White counterparts (Stephenson, 2001). Race-norming obfuscates the true prevalence of ADRD in Black patients and creates a significant barrier for Black patients to obtain sufficient dementia care.

Biomarker evaluations may provide a reprieve from implicit and racial bias in cognitive testing but are not without limitations. Previous studies have reported lower levels of cerebrospinal fluid biomarkers for ADRD in Black patients compared to White patients, including tau and phosphorylated tau (Howell et al., 2017; Morris et al., 2019). The patient’s race also impacted the interaction of apolipoprotein e4, a genetic biomarker for ADRD, and tau (Morris et al., 2019). Recently, a study also found that differences in tau levels for Black Americans can impair the accuracy of biomarker models predicting brain amyloidosis commonly used in ADRD evaluation (Schindler et al., 2022). These results led the authors to recommend future analyses of molecular ADRD biomarkers to adjust for race differences. Balls-Berry, authored on the article, proposes that methods for biological screening of ADRD need to be thought of “in terms of ancestry and in terms of social and structural determinants of health, which are often not looked at as being a biomarker for disease.”

## 4. Outreach and intervention programs for black women with ADRD

The disconnect between healthcare providers and Black patients can have a resounding impact on ADRD care. Black patients are twice as likely not to seek out healthcare when experiencing thinking or memory problems than White patients (Alzheimer’s Association, 2021). The reasoning behind the avoidance of healthcare intervention may be partly due to a lack of community educational outreach, as Black Americans are more likely to attribute ADRD symptoms to normal aging than White Americans (Glover et al., 2019). As cultural education among physicians remains subpar, these nuances in recognizing symptoms and treating different populations of patients impair the quality of care for ADRD (Assistant Secretary For Planning and Evaluation (ASPE), 2022). This divide widens as other factors such as socioeconomic status, level of education, and psychological stress not only inhibit access to high-quality dementia care but also put Black patients at greater risk for ADRD (Geronimus et al., 2010; Hill et al., 2015; Blair and Cybele Raver, 2016).

Historical mistrust of the medical community also impacts the likelihood that Black patients will seek out dementia care and other healthcare services (Green-Harris et al., 2019). Long-standing discrimination and ethical misconduct from medical and academic institutions toward Black Americans and minority communities have significantly damaged the relationship between patient and provider. Surveys indicate that 62% of Black Americans feel medical research is biased against people of color, and only 53% believe a cure for AD would be distributed fairly without discrimination (Alzheimer’s Association, 2021). Healthcare providers must first recognize and give credence to past misconduct and present mistrust in the relationship with Black patients. As Ball-Berry remarks, “What I would also like to see happen is for us to really acknowledge our historical hurts that we have done as institutions… and how that has further marginalized and impacted communities of color, especially the Black community, from being able to participate in studies because they felt like they were not heard when these things were happening.”

Lack of inclusion for Black patients in ongoing clinical research has limited scientific understanding and impacted the ability to generalize scientific findings and treatments to the larger community. “We have so much work to do— with less than 10% of African Americans being represented among Alzheimer’s clinical trials,” says Hill. Low recruitment of Black participants also occurs in complementary intervention programs and studies of ADRD risk factors (Alegria et al., 2021). Reliance on passive untailored recruitment strategies and implicit bias act as primary barriers to effectively reaching Black patients and underserved communities (Green-Harris et al., 2019). State-level efforts to rethink current outreach strategies have yielded key insights to the path forward for healthcare providers. The Wisconsin Registry for Alzheimer’s Project (WRAP) utilizes an asset-based community development approach that has improved access and care for Black patients with ADRD (Green-Harris et al., 2019). Their outreach efforts focused on identifying key community stakeholders, creating culturally-tailored programming, and providing educational resources for providers, patients, and caregivers. WRAP leaders concluded that by meeting the community’s needs first, Black patients were more likely to participate in ADRD research through WRAP.

Balls-Berry works to cultivate the same dynamic with patients for the Knight ADRC. She encourages “creating shared visions with our community and patient partners in a way that opens up doors for new places to recruit, to collect data, to provide health education, and showing up and giving our time to these communities because it’s going to advance the mission of those groups, not just our mission.” Knight ADRC partners with several organizations and key stakeholders in the community to facilitate outreach efforts toward Black patients and unserved communities. Similar efforts are underway at Southern Illinois University School of Medicine (SIUSOM), where the Smith Alzheimer’s Center has launched health equity initiatives through the Beyond the Medical Center Program in collaboration with the National Association for the Advancement of Colored People. SIUSOM also recently launched a Center for Equity in Professional Development to amplify community engagement and health education. Collectively, university ADRD outreach works to provide value to the community and encourage participation in clinical research.

Acting at the state and national level, Hill emphasized key takeaways that the Alzheimer’s Association encourages for outreach efforts. Ensuring cultural relevance, appropriateness, and effectiveness in all outreach materials and healthcare resources is paramount, according to Hill. The Alzheimer’s Association focuses on a community-based participatory model to accomplish this goal, emphasizing strategic partners in the community that are active at national and local levels, such as religious groups and healthcare workers organizations. Outreach efforts must go beyond just establishing community connections, but also listening and incorporating feedback from those partners. “We have to think critically and from an innovative perspective about how to get our free resources to the communities that are disproportionately affected, underrepresented, and underserved.”

## 5. Discussion

Black women seek help from a healthcare system that is fundamentally not built to serve them. From preventative care to delayed diagnosis and treatment, Black patients experience chronic discrimination inside and outside the healthcare system that contributes to a higher incidence of long-term health issues and places them at greater risk for ADRD. Implementing a training structure that actively recruits students from underserved communities and instills culturally competent care among healthcare providers is paramount to combating this issue. Such training must address implicit racial and ethnic bias and an understanding of the racial limitations of diagnostic tools. Further, more research is needed to understand the racial biases that may exist in ADRD biomarker and physiological evaluations of Black patients and to discover more culturally competent protocols for diagnosis. The diagnosis and treatment of ADRD require healthcare providers to become more aware of variations in the clinical presentation of ADRD by race and to customize their care and approach to their patients.

Improving dementia care for Black women also requires healthcare providers to connect with organizations and representative members of their communities. These members could serve as liaisons for communication that will help to build a trusting relationship within the Black community. Increasing access to healthcare services and education will also be required to help Black female patients to receive all-encompassing care. Following, the healthcare pathway prior to requiring ADRD care needs to improve. Establishing key contacts within the community can facilitate organizational strategies toward recruitment and dissemination of educational resources. A central tenet of any recruitment strategy must be to serve the community and provide preventative healthcare and education. By doing so, the organization can establish itself as a resource in the community and encourage patients to participate in clinical research. Underdiagnosis in this patient population may partially explain difficulties in recruiting for clinical trials. Increasing diversity in trial participation is a big push for both the Alzheimer’s Association and the NIA. The successful recruitment of more Black women to clinical research will aid in developing a deeper understanding of ADRD and facilitate the creation of more culturally competent dementia care for the future.

## Data availability statement

The original contributions presented in the study are included in the article/Supplementary material, further inquiries can be directed to the corresponding author.

## Author contributions

CF proposed the idea for the perspective article, conducted interviews, and acted as lead writer and project manager. MC assisted with project management, conducted interviews, and performed manuscript editing. AL and RB provided clinical thought partnership and revised the manuscript. KH, CY, and EH supervised project completion and revised the manuscript. All authors contributed to the article and approved the submitted version.

## Funding

This work was supported by the National Institutes of Health [NIA R01AG057767 and NIA R01AG061937], Dale and Deborah Smith Center for Alzheimer’s Research and Treatment, Kenneth Stark Endowment, and Meridian Health (CF, AL, RB, KH, EH).

## Conflict of interest

The authors declare that the research was conducted in the absence of any commercial or financial relationships that could be construed as a potential conflict of interest.

## Publisher’s note

All claims expressed in this article are solely those of the authors and do not necessarily represent those of their affiliated organizations, or those of the publisher, the editors and the reviewers. Any product that may be evaluated in this article, or claim that may be made by its manufacturer, is not guaranteed or endorsed by the publisher.
